# The Maze Pathway of Coevolution: A Critical Review over the *Leishmania* and Its Endosymbiotic History

**DOI:** 10.3390/genes12050657

**Published:** 2021-04-27

**Authors:** Lilian Motta Cantanhêde, Carlos Mata-Somarribas, Khaled Chourabi, Gabriela Pereira da Silva, Bruna Dias das Chagas, Luiza de Oliveira R. Pereira, Mariana Côrtes Boité, Elisa Cupolillo

**Affiliations:** Research on Leishmaniasis Laboratory, Oswaldo Cruz Institute, FIOCRUZ, Rio de Janeiro 21040360, Brazil; lilian.cantanhede@ioc.fiocruz.br (L.M.C.); carlos.somarribas@ioc.fiocruz.br (C.M.-S.); khaled.chourabi@ioc.fiocruz.br (K.C.); gabriela.silva@ioc.fiocruz.br (G.P.d.S.); bruna.chagas@ioc.fiocruz.br (B.D.d.C.); luizaper@ioc.fiocruz.br (L.d.O.R.P.); boitemc@ioc.fiocruz.br (M.C.B.)

**Keywords:** *Leishmania*, *Leishmania* viruses, phylogeny, coevolution, endosymbiont protozoan viruses

## Abstract

The description of the genus *Leishmania* as the causative agent of leishmaniasis occurred in the modern age. However, evolutionary studies suggest that the origin of *Leishmania* can be traced back to the Mesozoic era. Subsequently, during its evolutionary process, it achieved worldwide dispersion predating the breakup of the Gondwana supercontinent. It is assumed that this parasite evolved from monoxenic Trypanosomatidae. Phylogenetic studies locate dixenous *Leishmania* in a well-supported clade, in the recently named subfamily Leishmaniinae, which also includes monoxenous trypanosomatids. Virus-like particles have been reported in many species of this family. To date, several *Leishmania* species have been reported to be infected by *Leishmania* RNA virus (LRV) and *Leishbunyavirus* (LBV). Since the first descriptions of LRVs decades ago, differences in their genomic structures have been highlighted, leading to the designation of LRV1 in *L*. (*Viannia*) species and LRV2 in *L*. (*Leishmania*) species. There are strong indications that viruses that infect *Leishmania* spp. have the ability to enhance parasitic survival in humans as well as in experimental infections, through highly complex and specialized mechanisms. Phylogenetic analyses of these viruses have shown that their genomic differences correlate with the parasite species infected, suggesting a coevolutionary process. Herein, we will explore what has been described in the literature regarding the relationship between *Leishmania* and endosymbiotic *Leishmania* viruses and what is known about this association that could contribute to discussions about the worldwide dispersion of *Leishmania.*

## 1. Introduction

The origin of the *Leishmania* parasite dates back to the Mesozoic era, and models of its dispersion to the continents, still hypothetical, consider different scenarios [1]. The diversification of this group of dixenous parasites occurred on different continents, and currently, the *Leishmania* genus consists of dozens of different species worldwide, pathogenic to humans or not, which, by themselves, present complexities that are still not fully understood. There is some discussion on the taxonomy of *Leishmania,* and in this study, we will adopt the proposal of Kostygov et al. [2] and Espinosa et al. [3], naming four *Leishmania* subgenera: *L*. (*Leishmania*), *L*. (*Viannia*), *L*. (*Sauroleishmania*), and *L*. (*Mundinia*).

Despite efforts to unravel the mechanisms of *Leishmania* pathogenicity, to determine the risk of infection and to develop new treatments and vaccines against the parasite, there are still gaps in the state-of-the-art treatments to be explored. For example, one of the most well-defined aspects of the parasite, the *Leishmania* life cycle, has been updated by recent and important discoveries of factors that influence the parasite’s dispersion ability [4]. An amazing field to be explored concerns the effects of endosymbiotic *Leishmania* virus presence, its relationship with *Leishmania* cells, and further clinical and epidemiological consequences. *Paraleishmania hertigi* and *Paraleishmania deanei*, formerly *Leishmania hertigi* and *Leishmania deanei* [2,5], respectively, were the first members of the subfamily Leishmaniinae [6] identified as able to host virus-like particles [7]. Nevertheless, to date, no additional studies have been performed characterizing virus-like particles (VLPs) from these species. Only nine years later, *Leishmania (Viannia*) *guyanensis* and *Leishmania (Viannia*) *braziliensis*, which were both described as host viruses, were molecularly characterized [8], with the *L. guyanensis* virus named LR1 and the virus found in *L. braziliensis* named LR2. Both LR1 and LR2 were thought to contain single-stranded DNA [8], but soon after, they were demonstrated to contain circular double-stranded RNAs (dsRNAs) and were renamed *Leishmania* RNA virus (LRV) [9]. In the following years, LRV was described in 12 isolates of *L. braziliensis* and *L. guyanensis* from the Amazon region [10], and LRV was identified in one *Leishmania* (*Leishmania*) *major* isolate from a human patient in the former Soviet Union [11]. To the best of our knowledge, there are no studies that have searched for viruses in *L*. (*Sauroleishmania*) species, and only one species from *L*. (*Mundinia*), *L*. *martiniquensis,* was recently found to harbour a virus named *Leishbunyavirus* (LBV).

Although not much attention has been given to *Leishmania* viruses classification, altogether, the literature classified the viruses infecting *Leishmania* species, LRV and LBV, into two virus families: Totiviridae and Leishbuviridae, respectively. Recent reports focused on *Leishmania* and the viral endosymbiont LRV first arose from questions not directly related to the virus but rather to Toll-like receptors and their association with variable immunological responses to *Leishmania* infection [12]. Important data have been gathered since then. Both viruses, LBV and LRV, influence the phenotypic expression of *Leishmania* infection in the vertebrate host, although biological aspects of *Leishmania*-harbouring viruses vs. virus-free *Leishmania* remain to be elucidated.

Thus, current data, in association with reports from decades ago, led us a step further in the understanding of this peculiar, dynamic, and million-year-old parasite. Some studies were recently published searching for and characterizing viruses in *Leishmania* parasites and in different members of the Trypanosomatidae family, suggesting endosymbiotic viruses as an ancient acquisition by these protozoans. Here, our main objective is to summarize previous and recent reports that characterize *Leishmania* viruses and the impact of this endosymbiosis and then to analyse their relationships with the parasite species that host them.

## 2. The *Leishmania* viruses

Virus-like particles (VLPs) in parasitic protozoans were first described in *Entamoeba histolytica* in the 1960s [13]. After that, several studies reported similar structures in many unicellular eukaryotes, such as *Giardia lamblia*, *Trichomonas vaginalis*, and members of the Trypanosomatidae family, including *Leishmania* spp. and *Trypanosoma* spp. For some additional protozoans, however, there are only studies reporting VLPs based on electron microscopy approaches but not by molecular methods. The International Committee on Taxonomy of Viruses (ICTV) recognized only the family Totiviridae gathering *Leishmania* viruses [14]. However, this, as well as families of viruses collected from other trypanosomatids, must be updated considering recent virus discovery and characterization [15].

**Figure 1:**
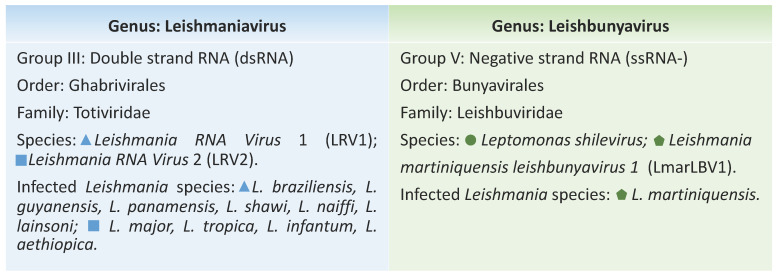
Classification of *Leishmaniavirus* and *Leishbunyavirus* viruses and *Leishmania* species described so far harbouring each of these endosymbionts.

Totiviridae consists of five genera: *Giardiavirus*, *Leishmaniavirus* (LRV), *Trichomonasvirus*, *Totivirus*, and *Victorivirus*. According to ICTV, *Leishmania* RNA virus 1 (LRV1) and *Leishmania* RNA virus 2 (LRV2) belong to the *Leishmaniavirus* genus. LRV was assumed to be capable of infecting *Leishmania* spp. only, with the two species identified as LRV1 and LRV2 (Figure 1), but recently, a member of this genus was also found in *Blechomonas* spp., a monoxenous trypanosomatid parasitizing fleas [15].

Recently, a new genus belonging to the Leishbuviridae family was proposed, *Leishbunyavirus* (LBV). The family Leishbuviridae includes the species *Leptomonas shilevirus*, which infects *Leptomonas moramango*, a monoxenic trypanosomatid [15], and LBV, which was found in *Leishmania martiniquensis* (Figure 1), a human pathogen that produces symptoms ranging from severe visceral disease to asymptomatic infections belonging to the subgenus *Leishmania* (*Mundinia*). The virus was denominated *Leishmania martiniquensis leishbunyavirus* 1 (*Lmar*LBV1) and represents the only non-LRV virus found to infect a *Leishmania* species so far [16]. The subgenus *L.* (*Mundinia*) has been established recently and remains understudied. It consists of newly emerging, human-infecting *Leishmania* species and nonhuman pathogens distributed worldwide. It has been assumed that this subgenus represents the earliest diverging branch within *Leishmania*, possibly transmitted by a different vector [17].

## 3. Exploiting Characteristics of *Leishmania*-Infecting Viruses

Leishmaniavirus species LRV1 and LRV2 were associated, respectively, with *Leishmania* (*Viannia*), found exclusively on the American continent, and with Old World *Leishmania* (*Leishmania*) species [18,19,20,21,22]. LBV, initially found in monoxenous trypanosomatids belonging to the subfamily Leishmaniinae and in the dixenous plant-parasitizing *Phytomonas* spp. [15], has also been detected in *Leishmania martiniquensis* [16] and possibly *Trypanosoma* spp. [15].

The Totiviridae family encompasses a broad range of viruses characterized by isometric virions, ranging from 30 to 40 nm in diameter, each containing a nonsegmented double-stranded RNA (dsRNA) genome, usually with two open reading frames (ORFs). LRV is a member of this family containing a ≅5.3 kb double-stranded RNA (dsRNA) genome [23,24] and organized into three ORFs. ORFs 2 and 3 encode a capsid protein (CP) and an RNA-dependent RNA polymerase (RdRP), respectively [25]. The first ORF is considered a predicted protein sequence and has shown no significant homology with known proteins [23]. Despite having small genomes, some totiviruses encode proteins in addition to RdRP and CP with known activity, such as the killer protein (KP4), produced by a fungal totivirus, which has proven antifungal activity [26]. Other totiviruses directly influence the expression of their host proteins, such as the virus that infects *Trichomonas vaginalis*, which, when present, is associated with an increase in the levels of proteins involved in the pathogenesis of the parasite [27]. Interestingly, although we do not know the protein encoded by ORF1 or its function, the viral capsid protein has endoribonuclease activity that precisely cleaves the transcript by ORF1 in both LRV1 [28] and LRV2 [29]. The two small RNA products resulting from the cleavage of their own endoribonuclease form a stable RNA/RNA complex, which can access host cell binding sites that are inaccessible to the transcript [30]. This configuration still requires further study. The classification of LRV in the Totiviridae family was due to its replication characteristics [31]. The low level of similarity (less than 40%) detected by comparing the nucleotide sequences from *L.* (*Viannia*) and *L. major* viruses enabled their classification into two different species, LRV1 and LRV2. Variation in the arrangement of the gene sequences is also observed between LRV1 and LRV2 [21,32]. LRV1 has an overlap between the regions encoding the viral capsid protein and the RNA polymerase, a particularity not observed for LRV2.

*Leishbunyavirus* belongs to the order Bunyavirales and is characterized as a virus exhibiting a negative-sense single-stranded RNA (ssRNA-) [33] organized in three genomic segments. The large segment encodes a viral RdRP, the medium segment encodes a surface glycoprotein precursor, and the small segment encodes a nucleoprotein [34]. Virions are usually 90 to 100 nm in diameter. The medium and small segments might present other ORFs involved in counteracting the host antiviral response, which may be present in both segments [35,36]. The infectivity and formation of viral particles in bunyaviruses depend on glycoproteins and type I transmembrane proteins that are proteolytically processed and glycosylated in the endoplasmic reticulum [36]. LmarLBV1 is a Bunyavirus and is the first non-LRV described infecting *Leishmania* [15,37].

Similar to other viruses, LRV and LBV require the resources of eukaryotic cells to sustain their metabolism. Furthermore, except for microRNAs (miRNAs) [38], dsRNA molecules are not produced by eukaryotic hosts, and eukaryotic cells have several mechanisms for detecting and inactivating these molecules [39,40]. The dsRNA viruses replicate within the capsid. Thus, the dsRNA genome is never exposed in the cytoplasm, which is an essential mechanism for evading host cell activation and antiviral action [41]. Transcription of the dsRNA genome by RdRP takes place within the virus [10]. The positive strand acts as messenger RNA (mRNA), giving rise to new viral particles, while the negative strand serves as a template for mRNA transcription [41].

## 4. A Brief History of the Detection and Dispersion of LRV1, LRV2 and LBV

LRV1 from the reference strain for *L*. *guyanensis* (MHOM/M4147) represents the first virus from kinetoplastids characterized by molecular approaches [8]. A few years later, the first study screening for the presence of LRV in *Leishmania* spp. strains from different geographical areas was conducted [42]. In this study, based on hybridization analysis, twelve LRV1 types (LRV1-1–LRV1-12) were defined, and it was shown for the first time that LRV1 could infect *L*. *braziliensis*, *L*. *guyanensis*, and various *Leishmania* strains from the Amazon Basin [42]. Comparative cDNA sequence analysis of LRV1-1 and LRV1-4 showed 77% identity, corroborating differences previously observed between these two types [32]. Furthermore, the comparison of two genomic regions from seven LRV types led to the description of two new types, LRV1-13 and LRV1-14, detected in *L*. *braziliensis* strains isolated from human patients from Bolivia [43].

In the early 1990s, parallel to the detection of LRV1 in two *L*. (*Viannia*) species, the discussion started as to whether the geographic distribution of *L*. (*Viannia*) spp. bearing LRV1 could be restricted to the Amazon Basin [42], despite widespread circulation of *L*. *braziliensis* in the American continent. Later, two other studies evaluated LRV1 in *L. braziliensis* from clinical samples and in *L. braziliensis* strains from south-eastern Brazil. All were negative [44,45], supporting the hypothesis of restricted circulation of *Leishmania* spp. bearing LRV1 to the Amazon Basin. Such findings exclude the possibility of a strict association between the presence of LRV1 and the severity of tegumentary leishmaniasis since there are also several leishmaniasis cases outside the Amazon Basin [20].

Recently, additional *L*. (*Viannia*) species were reported as infected by LRV1. Positive LRV1 samples were detected in tegumentary lesions from patients infected by *Leishmania (Viannia) lainsoni* and *Leishmania* (*Viannia*) *shawi* living in the western Brazilian Amazon region [20]. Later, LRV1 was demonstrated and characterized in the reference strain of *L. shawi* (MCEB/BR/1984/M8408), a strain isolated from a monkey [46]. A survey aiming to detect LRV in *Leishmania* strains deposited at the *Leishmania* collection of the Fundação Oswaldo Cruz-CLIOC. Available online: http://clioc.fiocruz.br (accessed on 6 April 2021) is underway and it was detected LRV1 in another *L. shawi* strain isolated from a human patient presenting CL in the Amazonas state (Table S1). An *Leishmania* (*Viannia*) *naiffi* strain from Amazonas state in Brazil was also reported to be *positive* for LRV1 [47] and 11 *L. naiffi* strains deposited at CLIOC were also positive, as well as one *L*. *lainsoni* (Table S1), the latter corroborating our previous study detecting LRV1 in clinical samples from patients infected by this species. All aforementioned results corroborated the assumption that LRV1 is restricted to *Leishmania* strains circulating in the Amazon Basin. However, we cannot rule out that the apparent narrow geographical distribution of LRV1 might be a result of biased surveys. Studying *Leishmania* spp. from Costa Rica, we detected an *L*. *guyanensis* strain positive for LRV1 (Table S1), reinforcing a recent finding indicating the circulation of LRV1 in this area [48].

In 1993, a virus was identified in an Old World *Leishmania* species, *L. major*, and was designated LRV2-1. It was described as immunologically distinct when compared to LRV1-1 and LRV1-4 [11]. The complete sequence of the virus found in *L*. *major* promastigotes MHOM/SU/1973/5-ASKH was published two years later, and it showed that the most relevant characteristic distinguishing the genomic structure of LRV2 from LRV1 and other totiviruses is the nonoverlapping capsid and RdRP genes [21].

LRV2 was detected in *L. major* [21], *Leishmania infantum* [22], *Leishmania aethiopica* [18,49], and *Leishmania tropica* [50]. Two studies conducted in Iran, in a zoonotic focus of cutaneous leishmaniasis (CL) and including visceral leishmaniasis (VL) patients, reported that the virus was detected in two different parasite specimens: one *L*. *infantum* strain derived from a VL patient unresponsive to treatment using meglumine antimoniate and one *L*. *major* strain from a great gerbil, *Rhombomys opimus* [22]. More recently, a survey was conducted in isolated promastigotes from 85 CL human patients from Iran. Eighty-three were identified as *L*. *major* and 2 as *L*. *tropica*. Fifty-nine (69.4%) presented LRV2, and one out of the two *L*. *tropica* isolates was also positive for LRV2 [50]. *L*. *tropica* was first demonstrated to be infected by LRV2 in a survey conducted in Turkey, in which 7 LRV2-positive *L*. *tropica* strains out of 24 were identified [51].

Recently, LRV2 was described in three (out of 3 examined) *L*. *major* strains in Turkey [51] and in two *L*. *major* strains isolated from CL patients from Uzbekistan. Sequence analysis indicated a high similarity between the two LRV2 isolates from Uzbekistan, which were closely related to the LRV2 isolate found in the *L*. *major* strain ASKH documented in Turkmenistan [21,49]. Thus, the presence of LRV2 in *L*. *major* is possibly frequent and widespread.

Recently, for the first time, a study demonstrated *L*. (*M*.) *martiniquensis* infected by endosymbiotic virus, a *Leishbunyavirus* (LBV). The molecular characterization revealed a genomic arrangement with three segments and sequences similar to those of LBV, which was first described infecting monoxenous *Crithidia* spp., a trypanosomatid member of the subfamily Leishmaniinae. However, to the best of our knowledge, the work published by Grybchuk and colleagues in 2018 [15] presented the most comprehensive study on LBV. In summary, thus far, LBV represents the most widespread and species-rich group of RNA viruses from trypanosomatids. This virus was found in *Crithidia* spp. from Ecuador, Ghana, and Russia and *L. moramango* from Madagascar, monoxenous trypanosomatid strains isolated from different hosts. Furthermore, using metatranscriptomic data from dipterans and horse leeches for viral and trypanosomatid surveys, they proposed this group of viruses associated with the subfamily Strigomonadinae and with *Trypanosoma* spp.

Regarding *Leishmania*, it is interesting that a geographically dispersed and multiple-host virus was detected in *L*. (*Mundinia*) species, the earliest branch within the genus *Leishmania*, which likely originated before Gondwana’s breakup [52,53]. Another interesting feature of this group of parasites is concerned with its geographical dispersion and the diversity of vertebrates implicated as hosts, including humans. Interestingly, this group of parasites is probably not transmitted by sandflies. Comparative genomic analysis shows interesting differences in *L. (Mundinia)* from other *Leishmania* species [17].

## 5. LRV and LBV Modulating *Leishmania* spp. Phenotypes

*Leishmania* spp. infected with either Leishbunyaviridae or Totiviridae viruses show altered phenotypic expression. Several studies have been proposed to understand this impact, mainly involving the LRV1 endosymbiont, on the biology of different *L*. (*Viannia*) strains. The reason for this might be the enigmatic pathophysiology of CL and the intriguing hypothesis that LRV confers either a state of hypovirulence or hypervirulence on the host-parasite interaction [54].

Several groups have speculated on the influence of LRV1 on parasite virulence, and years have passed without major studies on the biological impact of LVR1 on *Leishmania* parasites [55]. Concern about LRV1 as a determinant of parasitic virulence reappeared in a 2011 study by Ives and colleagues using clones of *L*. *guyanensis* clinical isolates. Samples were classified due to their tendency to metastasize, ranging from highly metastatic (M+) to nonmetastatic (M−), using hamsters as the animal model. The authors found that a mucosal lesion-associated clone *L*. *guyanensis* carrying the virus (LgM+) increased the endogenous immune response in an unregulated manner, promoting an increase in inflammatory cytokines. These clones resulted in a phenotype of severe destruction of the nasopharyngeal mucosa when inoculated in mice, despite the significant reduction in the number of parasites. Macrophages infected with virus showed a phenotype similar to macrophages infected with parasites (LgM+), with increased expression levels of chemokines and cytokines such as CXCL10, CCL5, tumour necrosis factor-Alpha (TNF-α), and interleukin 6 (IL-6), also demonstrating that LRV1 alone induced the intensification of the inflammatory response to *Leishmania* antigens [12].

Thereafter, several studies explored the participation of LRV in the clinical evolution of the disease. Our group demonstrated that the relative risk of developing mucosal lesions in patients with Tegumentary leishmaniasis and LRV1 was three times higher than that in patients infected with parasites without LRV1 [20]. Moreover, the presence of LRV1 was associated with therapeutic failure cases in patients infected with *L*. *guyanensis* [56] and in patients infected with *L*. *braziliensis* [57]. However, other reports did not correlate LRV with distinct clinical phenotypes of TL [44,54] or treatment failure [58,59].

Assuming a mutualistic relationship between LRV and *Leishmania* spp., it is expected that *Leishmania* harbouring LRV1 could display better performance and fitness than virus-free strains facing certain environmental challenges. Routine evaluation of cultures maintained at CLIOC indicates two patterns of growth among *L. guyanensis* strains, and it was observed that LgLRV1+ survived longer and despite the environmental stress faced by parasites during in vitro cultivation, maintains viable parasites even in a nutrient-depleted environment without medium replacement (Figure S1) [60]. The reference strain for *L. guyanensis* (MHOM/BR/1975/M4147) is LRV1+, and a previous study demonstrated the detection of viable parasites until the end of the monitoring of the culture [61].

Studies have reported data on LRV+ and LRV− parasites under the same environment, for example, growing in the same culture medium [12,62], although there are apparently always fewer negative than positive parasites [63], suggesting that few LgLRV1− parasites may remain viable for a long time when cocultivated with LgLRV1+ parasites. We observed that experimentally mixed LgLRV1−/LgLRV1+ cultures presented a similar number of viable parasites at day 9 to that observed in single cultures for the LgLRV1+ strain (Figure S1), suggesting either (i) the counted parasites corresponded strictly to LgLRV+ cells or (ii) cocultivation enhances LVR− parasites’ ability to survive. However, it is plausible that *Leishmania* spp., as described in *Trypanossoma brucei* [64], synthesize and secrete compounds in the shared environment, affecting population density and parasite behaviour, measured, for example, by growth rate in culture. It is possible that in addition to mechanisms such as cell-cell contact and secretion factors, exosome secretion, recently demonstrated for LRV1+ parasites, also contributes to this interaction [65,66,67].

Studies using mice infected by *L*. *guyanensis* LRV1+ demonstrated a higher parasite burden in lesions produced by these parasites than those produced by *L*. *guyanensis* LRV1− [12,68,69]. The immunization of mice with a vaccine produced from the LRV1 viral capsid protein decreases the burden of parasites in lesions after a new infection with *L*. *guyanensis* LRV1+ [68].

Little information is available concerning the influence of LRV on the biology and gene expression of *Leishmania* parasites when infected by these viruses. Teleologically, the viruses might influence the expression of many *Leishmania* genes, not only, but mainly those influenced by stressful conditions generated by parasite proliferation. Bearing in mind characteristics of infections caused by parasites containing LRV1, genes implicated on parasite proliferation and persistence are good target to be investigated also. Not less important are genes associated to therapeutic failure in infections caused by *Leishmania* parasites, pondering that cases of therapeutic failure have been associated with the presence of LRV1 in patients infected by *L*. *braziliensis* [57] and by *L*. *guyanensis* [56].

LRV is found in both stages of the *Leishmania* life cycle: promastigotes and intracellular amastigotes [8,9]. However, despite several studies exploring the effect of LRV in leishmaniasis pathogenesis, it is still unclear whether the virus effect is either the response of the vertebrate host to viral infection or if the virus affects the biology of its own host, *Leishmania* spp. [55,69]. A recent study evaluated the effect of LRV1 on the pathogenesis of TL using an isogenic, high viral load clone of *L*. *guyanensis* LRV− (from the M4147 strain). In doing so, it was possible to evaluate the effect of the virus in inducing the innate immune response. This study deciphered the mechanism by which LRV1 promotes parasitic persistence and disease progression and showed that this occurred due to the limited activation of inflammasomes in macrophages. Such an effect of LRV1 in modulating the immune response has also been demonstrated in human samples and was associated with mucosal leishmaniasis [70]. Additionally, as already mentioned, the presence of LRV1 and the viral load were identified as crucial factors in disease severity and pathology [62]. However, a question remains regarding the participation of LRV1 in modulating the immune response: it has been shown that the virus can be transported via exosomes [66], but at what point of infection is LRV1 exposed to the host cell, signalling the cascade that leads to the most severe phenotype of the disease?

Like LRV1, LRV2 present in *L*. *aethiopica* strains isolated from humans (LRV2-Lae) showed potential in modulating the immune response in macrophages, resulting in a hyperinflammatory and TLR3-dependent response [18]. In *Leishmania tropica*, LRV2 was detected in approximately 30% of the strains analysed [51]. *L. tropica* is an important aetiological agent of cutaneous leishmaniasis in the Old World, and there are several reports of this species in cases of mucosal leishmaniasis [71,72,73,74].

In Ethiopia and Brazil, a portion of patients with cutaneous lesions commonly progress to severe forms of the disease, such as mucosal leishmaniasis [75]. In those cases, the presence of LRV was associated with the development of the mucosal phenotype.

Despite the common influence of both LRV types on the immune response, other characteristics were not shared between them. For example, the LRV2 present in *L*. *major* isolates did not affect the therapeutic response [58], as already reported in infections by *L*. *guyanensis* and *L*. *braziliensis* LRV1+ [56,57]. However, a report of *Leishmania infantum* harbouring LRV2 described a patient with visceral leishmaniasis who had not responded to three cycles of systemic treatment. Therefore, not enough evidence is available to associate the presence of LRV2 with clinical phenotypes in VL caused by *L*. *infantum* [22].

The LBV detected in *L*. *martiniquensis* (LmarLBV1) also seems to influence parasite pathogenicity. Using an isogenic clone of *L*. *martiniquensis* without LBV (LmarLBV1-depleted), the influence of the virus on the biology of the parasite was evaluated, specifically concerning its ability to infect murine macrophages. The results showed that the LmarLBV1-depleted strain was less infective than the LmarLBV1 strain, indicating that LmarLBV1 facilitates parasite infectivity in vitro in the primary murine macrophage model [16].

## 6. The Maze Pathway of Coevolution of *Leishmania* spp. and Its Viruses

It is not yet fully known how *Leishmania* viruses are maintained and transmitted to *Leishmania* parasites. The most common mechanism for viral transmission in the Totiviridae family may be either vertical, horizontal (by cell fusion), or both, propagation [76]. Infection of non-LRV1-infected *Leishmania* parasites failed or was transitory when electroporation was attempted [77]. Mature viral particles of LRV could be transmitted to new parasites by cell division [11,41] or via exosomes [66]. More than 30% of exosomes produced by an *L*. *guyanensis* strain carry viral particles, and inside exosomes, LRV1 is able to resist inhospitable conditions until exosome-enveloped LRV1 infects other parasites [66]. Extracellular transmission of Totivirus in some protozoan parasites, such as *Giardia lamblia* [78], and in *L. guyanensis* via exosomes [66] has been documented. Although this transmission is probably rare, virus-infected and noninfected parasites are still observed in the same culture [63]. It could not be ruled out that some parasites are resistant to virus infection, a hypothesis that remains to be tested.

The lack of a detectable infectious phase of LRV suggests a long-lasting relationship between the virus and the parasites, representing a symbiotic association. Indeed, studies have shown similar genetic intervals between *Leishmania* species and LRV1 and LRV2 [21,43]. Phylogenetic findings suggested that LRV acquisition by *Leishmania* parasites occurred prior to the divergence of Old and New World *Leishmania* parasites [43], but its interaction with the Trypanosomatid family was ancient, as indicated by the finding of LRV in *Blechomonas* [15].

**Figure 2:**
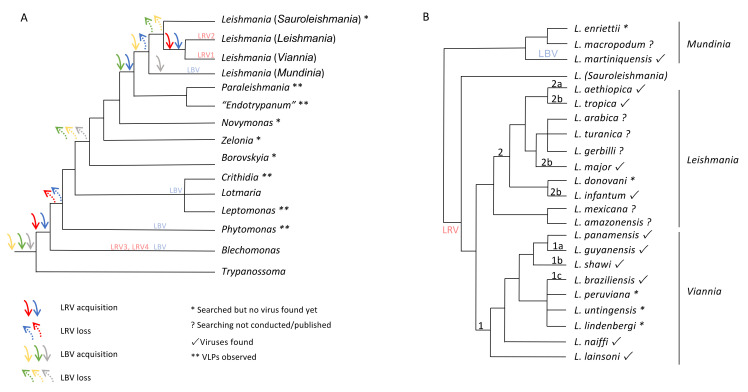
Schematic phylogenetic tree for the family Trypanosomatidae (**A**) and the genus *Leishmania* (**B**) based on published data [2,3,15] showing possibilities of *Leishbunyavirus* (LBV) and *Leishmania* RNA virus (LRV) acquisition by members of the family Trypanosomatidae and LRV dispersion across *Leishmania* species. Three scenarios are possible for LBV (green, yellow and grey arrows): green—ancient acquisition, with possible loss (dashed green arrow) in the first Leishmaniinae split and new acquisition in the clade containing *Leishmania*, *Paraleishmania*, and “*Endotrypanum*”, followed by loss in the split of *Leishmania* (*Mundinia*) from the other three *Leishmania* subgenera. This scenario assumes LBV not infecting *Novymonas*, *Zelonia* and *Borovskyia* and virus-like particles (VLPs) found in the clade containing Paraleishmania and “Endotrypanum” as LBV; yellow—the same as green, but with the last acquisition occurring in the split of *L*. (*Mundinia*) from the other *Leishmania* subgenera and subsequent loss in members of the other three *Leishmania* subgenera, assuming VLPs found in the clade Paraleishmania and “Endotrypanum” are not LBV; grey—ancient with possible loss (dashed grey arrow) in the first Leishmaniinae split and new acquisition when *L*. (*Mundinia*) split from the other *Leishmania* subgenera. Scenarios expected for LRV: blue—acquisition by a monoxenous trypanosomatid followed by sequential loss when another dixenous clade appears and acquisition in the clade containing *Leishmania*, Paraleishmania, and “Endotrypanum”, followed by loss when *L*. (*Mundinia*) split from the other *Leishmania* subgenera and a new acquisition by clade *L*. (*Viannia*)/*L*. (*Leishmania*); this scenario assumes VLPs found in the clade containing Paraleishmania and “Endotrypanum” are LRV and the possibility of LRV infecting all *Leishmania* subgenera; red—acquisition by a monoxenous trypanosomatid followed by sequential loss when another dixenous clade appears and a new acquisition by clade *L*. (*Viannia*)/*L*. (*Leishmania*).

Viral particles, LBV and LRV, were found in *Leishmania* species and in their closest phylogenetic clades *Endotrypanum* spp. and *Paraleishmania* spp. The loss and acquisition of both LBV and LRV probably occurred early in the family Trypanosomatidae, but additional research with different specimens from this family is necessary to make a proper inference for this hypothesis. Considering the knowledge gathered so far, the relationship between LBV and members of the Trypanosomatidae is older than that observed for LRV. LBV appears in *Trypanosoma* spp., regardless of whether *Blechomonas* is the first genus of the family harbouring LRV. LBV was detected in several members of the Trypanosomatidae family [15]. Although LRV, more specifically LRV3 and LRV4, was observed in *Blechomonas*, prior to the moment when Leishmaniinae split from other trypanosomatids, this virus emerged again in the *Leishmania* spp. branch. This could have coincided with the point in time when the dixenous life cycle emerged in Leishmaniinae, which could be supported by the identification of VLPs in *Paraleishmania* and *Endotrypanum* [2] as LRV, although a characterization of these particles is still required. Another possibility that can be assumed is the re-emergence of LRV before the time of *L*. (*Viannia*) and *L*. (*Leishmania*) diversification, considering VLPs found in *Paraleishmania* and *Endotrypanum* as non-LRVs. Comparative analyses of the *Leishmania* tree, based on random amplified polymorphic DNA (RAPD), and the LRV trees, obtained by sequence analysis of ORF3 or the 5′ untranslated region (5′-UTR), supported a long history of coevolution between LRV and the parasite-host strains, sustaining the hypothesis that LRV is an ancient virus of *Leishmania* spp. [43] and probably spread following host diversification (Figure 2).

Phylogenetic studies have shown that the transition from a monoxenous to a dixenous state occurred at least three times in the family Trypanosomatidae, giving rise to parasites of vertebrates, such as the *Trypanosoma* and *Leishmania* genera, and to *Phytomonas*, a dixenous phytopathogenic genus. Therefore, monoxenous parasites of invertebrates were ancestors of dixenous pathogens [79]. Considering the phylogenetic reconstruction of viruses found in many trypanosomatids using RdRP sequences, a well-supported clade for LBV was observed to be closely related to Phenuiviridae [15], a family including many viruses from insects, including the genus *Phlebovirus*, which is transmitted by sandfly species, the *Leishmania* vectors [80].

Assuming monophyly in the *Leishmania* clade and including their sister clades *Endotrypanum* spp. and *Paraleishmania* spp., two different points in time appear when the acquisition of these viruses could have occurred: first for the LBV in the subgenus *L*. (*Mundinia*) and then for the LRV in *L*. (*Viannia*) and *L*. (*Leishmania*), with a later diversification into LRV1 and LRV2 at the same time that these two *Leishmania* subgenera split [18,43]. The challenge is now to uncover the points when gain and loss of the viruses appear in the process of diversification of the trypanosomatid taxa. Different strains from the same taxon can be found infected and noninfected by a specific virus, but it is still unknown whether the virus infection is an ancestral character or a derived one. The common ancestor for the *Leishmania* clade and their sister clades *Endotrypanum* spp. and *Paraleishmania* spp. could be virus-free, and independent viral acquisitions could have subsequently occurred. Different routes of both LBV and LRV acquisition and loss are possible in this protozoan group considering data gathered so far (Figure 2).

Alternatively, virus loss might have occurred independently and randomly. For strains from the same species, it is plausible that a given strain, or its ancestor, was infected, and during binary division, the virus was not equally transferred, resulting in both infected and noninfected descendants. This hypothesis also explains the observation of virus-infected and noninfected parasites in the same culture. To explore such an alternative, we consider LRV1 and *L*. (*Viannia*) as an example. LRV1 was detected in most of the *L*. (*Viannia*) species: *L*. *guyanensis*, *L*. *braziliensis*, *L*. *shawi*, *L*. *naiffi*, *L*. *lainsoni*, and *Leishmania panamensis*. Sequence analysis of LRV1 from *L*. *braziliensis*, *L*. *guyanensis*, and *L*. *shawi* showed clusters gathering according to the *Leishmania* species (Figure 2); the sole LRV1 sequence analysed from *L*. *shawi* was placed among two LRV1 sequences from *L*. *guyanensis* [46,63]. Curiously, the similarity between *L. shawi* and *L*. *guyanensis* was reported in many studies [81,82,83] and was also detected when LRV1-*L*. *guyanensis* and LRV1-*L. shawi* sequences were analysed [46]. Microsatellite analysis of *L*. (*Viannia*) spp. indicated that *L*. *guyanensis* is a distinct population within the *L. (Viannia*) subgenus (by microsatellite analysis), with no distinguishable subpopulations. However, differences in the reactivity profile with monoclonal antibodies were detected, overlapping the geographical distribution of the strains [84,85] and correlating with clusters formed after LRV1 *L*. *guyanensis* sequence analysis [46].

The case of *L*. *braziliensis* is especially interesting, as this species is widespread in the American continent, but so far, LRV1 has been detected only in strains isolated from the Amazon region. By microsatellite analyses, LRV(−) *L*. *braziliensis* strains belong to a distinct population from LRV1-infected *L*. *braziliensis* [86,87]. The intragroup diversity detected by the *L*. *braziliensis*-LRV1 sequence analysis is as high as the heterogeneity reported for this parasite species [88,89,90]. Two LRV1 clusters were demonstrated, corresponding to *L*. *braziliensis* from the western Amazon region (one from Bolivia and one from Brazil); an *L*. *braziliensis*-LRV1 sequence from French Guyana was placed in the middle, but with lower bootstrap support [46].

For *L*. *guyanensis*, *L*. *braziliensis*, other *L*. (*Viannia*) spp., and species infected by LRV2, infected and noninfected parasite cells were detected within the same strain. The same occurs for strains from the same regions. This assortment might be a significant determinant of coevolution [91] assuming that the degree of mixing, virus-free and virus-infected *Leishmania* spp. would increase *Leishmania* spp. exposure to viruses, therefore selecting for greater resistance and infectivity intervals. The characteristics of *Leishmania* and LRV could influence the probability of fluctuation in the direction of natural selection for a given phenotype over an evolutionary period of time (fluctuating selection dynamics—FSD). Furthermore, it could also be possible that *Leishmania* and their viruses are in combat, causing both to select adaptive characteristics, leading them to coevolve (arms race dynamics—ARD). The shift from FSD to ARD associated with population mixing is a possibility to be acknowledged [91]. Considering the infection by LRV in *Leishmania* species since the diversification of the subfamily Leishmaniinae, ARD could explain the lack of LRV-infected *L. braziliensis* outside of the Amazon Basin. If this is the case, *L. braziliensis* and LRV1 developed different resistance and infectivity (or strategies of infection), respectively. The raised hypothesis assumed the existence of *L. braziliensis* populations resistant to LRV infection. The methodology used to describe LRV transmission via exosomes [66] to uninfected *L. guyanensis* could be applied to test this assumption.

It seems that *L. braziliensis* parasites without LRV1 have been better adapted to the conditions encountered, especially in relation to the phlebotomine species, indicating that a bottleneck phenomenon occurred during the spread of *L*. *braziliensis*. Considering microsatellite analyses, there is one *L*. (*Viannia*) population in the Amazon region consisting of *L*. *braziliensis* strains and other *L*. (*Viannia*) species—*L*. *guyanensis* excluded. This diverse population is organized into subpopulations that match species identity [87]. In previous studies [20,43,47,64] and corroborated by the presented data, LRV1 infection was described in many *L*. (*Viannia*) species. It remains unsolved, however, whether LRV1 from these species is related to *L*. *braziliensis* LRV1. To address many of the points raised, it is important to conduct phylogenetic studies of LRV1, LRV2, LBV, and their hosts. It is noteworthy that the phylogenetic trees for LRV [43] and LBV [15] display congruence with those obtained for their hosts, suggesting coevolution and limitation of horizontal viral transmission.

## 7. Concluding Remarks

The hypothesis that parasites influence the population size or geographical dispersion of their host is opposed by a more acceptable hypothesis arguing that successful or well-adapted parasites evolve to be harmless to their host. Although virus-like particles and viruses were first detected in *Leishmania* parasites some decades ago, the impact of this interaction and the diversity of these endosymbionts have recently drawn considerable attention, mainly due to the virulence trade-off experimentally demonstrated in the context of *Leishmania* (*Viannia*) *guyanensis* and LRV1. However, the theory regarding the evolution of interactions among different endosymbiotic viruses and *Leishmania* spp. is still in its emerging stages. Recent studies have reported the discovery of several viruses in trypanosomatids, indicating the existence of unknown viral diversity, which needs to be further investigated and can provide important evolutionary information. At least two virus families have already been described as *Leishmania* spp. endosymbionts, but we still do not know if these viruses occur only in *Leishmania* spp. or if they can be detected elsewhere, such as in the invertebrate host of *Leishmania* spp. It is plausible to assume a dynamic symbiotic relationship in this long-term interaction between LRV or LBV and *Leishmania* spp., but the influence of either LRV or LBV on *Leishmania* biology is not yet clear. At least in some circumstances, it seems that this interaction causes a stressful condition, promoting increased tolerance of *Leishmania* spp. to some environments and augmenting its replication rate. It is a fact that both viruses influence leishmaniasis pathogenesis, but it is still unclear whether this is a consequence of the vertebrate host response to the virus living in *Leishmania* spp. cytoplasm or a biological response of *Leishmania* spp. to the endosymbiotic viruses. The impact of a “parasitized parasite” in the initial moments of a natural infection is also an aspect that deserves attention. The phenotype of higher pathogenicity linked to *Leishmania* spp. bearing viruses might be linked to an increased evolutionary fitness might be considered to signal that viral acquisition was beneficial to the parasite. However, there is also the possibility of better fitness for those organisms that are less pathogenic, which could have the chance to produce asymptomatic infections, to be maintained longer in the vertebrate host, and to be dispersed to new environments.

The screening of viruses in *Leishmania* spp. is still limited to a few studies, but so far, the evidence has indicated that LRV1 is restricted to the American continent and associated with *Leishmania* (*Viannia*) species and that LRV2 is linked to the Old World and hosted by *Leishmania* (*Leishmania*). LBV was detected only in *L*. *martiniquensis*, a species belonging to a subgenus not closely related to *L*. (*Viannia*) or *L*. (*Leishmania*). For both LRV1 and LRV2, there were different genotypes and correlations with the parasitized *Leishmania* species. The consequence of *Leishmania*-LRV or *Leishmania*-LBV coevolution was probably dependent on coevolutionary dynamics, involving (i) fluctuating selection affecting the frequency of some genotypes, especially those linked to resistance and infectivity [92] or fluctuations in the ranges of resistance and infectivity [93], and (ii) antagonist coevolution turning towards either increasing infectivity, resistance, or both. There is an important imbroglio of evolution and ecology linked to the relationship between *Leishmania* spp. and LRV or LBV, these interactions providing a direct impact on the evolutionary route.

## Data Availability

Not applicable.

## Supplementary Materials

The following are available online at https://www.mdpi.com/article/10.3390/genes12050657/s1, Supplementary Data 1: LRV1 detection in *Leishmania* spp. Table S1: Strains of *L*. (*Viannia*) positive for LRV1. Supplementary Data 2: Growth curve of *Leishmania guyanensis* strains infected or not with the *Leishmania* RNA virus in single or mixed culture. Figure S1: Growth curve of *Leishmania guyanensis* strains infected or not with the *Leishmania* RNA virus in single or mixed culture.
