# Supplementary material for: The Maze Pathway of Coevolution: A Critical Review over the Leishmania and Its Endosymbiotic History
Source: Genes (Basel). 2021 Apr 27;12(5):657. doi: 10.3390/genes12050657 (PMC8146029; doi:10.3390/genes12050657)
Supplement: Supplementary file 1 [file genes-12-00657-s001.zip › genes-1094199-supplementary/Supplementary Material.pdf]

Supplementary Material

Supplementary Data 1:

LRV1 detection in *Leishmania* spp.

LRV1 detection in *Leishmania* spp strains deposited at the *Leishmania* Collection of the Fundação Oswaldo Cruz (CLIOC) followed the protocol described by Cantanhêde et al. [1,2]

Table S1. Strains of *L.* (*Viannia*) positive for LRV1.

ID	*Leishmania* Strain ID	Parasite Specie	*Leishmania* International Code	Geographic Origin
1	IOCL 3316	*Leishmania naiffi*	MHOM/BR/2011/58-AMS	Pará/BR
2	IOCL 3515	*Leishmania naiffi*	MHOM/BR/2013/49UAS	Amazonas/BR
3	IOCL 3516	*Leishmania naiffi*	MHOM/BR/2013/63DDL	Amazonas/BR
4	IOCL 3517	*Leishmania naiffi*	MHOM/BR/2013/65HCC	Amazonas/BR
5	IOCL 3518	*Leishmania naiffi*	MHOM/BR/2013/66CPS	Amazonas/BR
6	IOCL 3519	*Leishmania naiffi*	MHOM/BR/2013/51FRS	Amazonas/BR
7	IOCL 3520	*Leishmania naiffi*	MHOM/BR/2013/62FJFM	Amazonas/BR
8	IOCL 3523	*Leishmania naiffi*	MHOM/BR/2013/25EPF	Amazonas/BR
9	IOCL 3524	*Leishmania naiffi*	MHOM/BR/2013/45JOM	Amazonas/BR
10	IOCL 3525	*Leishmania naiffi*	MHOM/BR/2013/56EGP	Amazonas/BR
11	IOCL 3574	*Leishmania naiffi*	MHOM/BR/2015/352 FMS	Amazonas/BR
12	IOCL 1545	*Leishmania shawi*	MCEB/BR/1984/M8408	Pará/BR
13	IOCL 3481	*Leishmania shawi*	MHOM/BR/2013/18 LTA MLF	Amazonas/BR
14	IOCL 3398	*Leishmania lainsoni*	MHOM/BR/2012/AP60A	Rondônia/BR
15	IOCL 3804	*Leishmania guyanensis*	MHOM/CR/2019/108-GML	Costa Rica

Supplementary Data 2:

Growth curve of *Leishmania guyanensis* strains infected or not with the *Leishmania* RNA Virus in single or mixed culture

Experimental analysis of the growth rate comparing two *L. guyanensis* strains: IOCL 3357; LgLRV1+ and IOCL 3521; LgLRV1- was performed using the protocol previously described [1,2]. Growth curve of a mixed culture containing LgLRV1+ and LgLRV1- started with 0.5 x 10^6 parasites/ml of each strain .

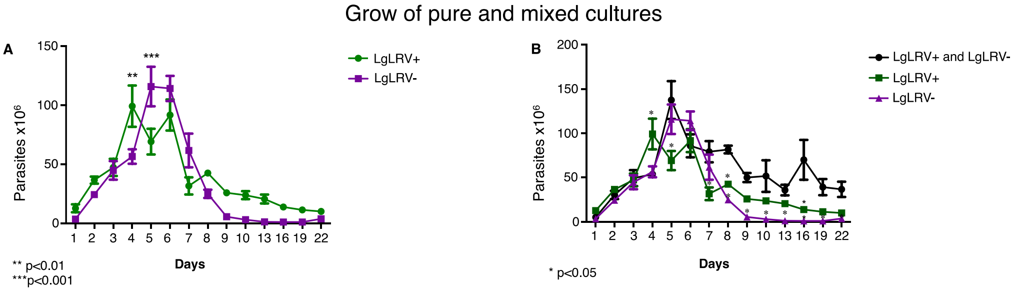

Figure S1. Growth curve of *Leishmania guyanensis* strains infected or not with the *Leishmania* RNA Virus in single (A) or mixed culture (B). *L. guyanensis* strains were cultivated in Schneider´s medium (Vitrocell Embriolife, Campinas, SP, BR) supplemented with 20% fetal bovine serum (FBS) (Vitrocell Embriolife, Campinas, SP, BR), starting with 1x10^6 parasites/ml for single cultures or 0.5x10^6 parasites/ml of each for mixed cultures. Strains employed: *L. guyanensis* LRV1 positive (LgLRV+) - MHOM/BR/2011/S83 and *L. guyanensis* LRV negative (LgLRV-) - MHOM/BR/2013/57FRMS.

NOTES: LgLRV1+ survived longer than LgLRV1-. The *L. guyanensis* LRV1 negative strain showed a similar profile to that of the LgLRV1+ until the 7th day of culture,

although with a greater number of parasites on the 5th and 6th days of culture. However, on the 7th day, the number of parasites declined, and after the 9th day, no viable parasites were detected. A study that evaluated the reference strain for *L. guyanensis* (MHOM/BR/1975/M4147), LRV1+ also detects viable parasites until the end of the monitoring of the culture [3].

References

1. Cantanhêde, L.M.; da Silva Júnior, C.F.; Ito, M.M.; Felipin, K.P.; Nicolete, R.; Salcedo, J.M.V.; Porrozzi, R.; Cupolillo, E.; Ferreira, R. de G.M. Further Evidence of an Association between the Presence of Leishmania RNA Virus 1 and the Mucosal Manifestations in Tegumentary Leishmaniasis Patients. *PLoS neglected tropical diseases* **2015**, *9*, e0004079, doi:10.1371/journal.pntd.0004079.
2. Cantanhêde, L.M.; Fernandes, F.G.; Eduardo Melim Ferreira, G.; Porrozzi, R.; De Godoi Mattos Ferreira, R.; Cupolillo, E. New insights into the genetic diversity of Leishmania RNA Virus 1 and its species-specific relationship with Leishmania parasites. *PLoS ONE* **2018**, *13*, 1–16, doi:10.1371/journal.pone.0198727.
3. Mendes, B.P.; Da Silva, I.A.; Damata, J.P.; Castro-Gomes, T.; Vieira, L.Q.; Ribeiro-Dias, F.; Horta, M.F. Metacyclogenesis of Leishmania (Viannia) guyanensis: A comprehensive study of the main transformation features in axenic culture and purification of metacyclic promastigotes by negative selection with Bauhinia purpurea lectin. *Parasitology* **2019**, 716–727, doi:10.1017/S0031182018002111.
