# Supplementary figures and images for: The Maze Pathway of Coevolution: A Critical Review over the Leishmania and Its Endosymbiotic History
Source: Genes (Basel). 2021 Apr 27;12(5):657. doi: 10.3390/genes12050657 (PMC8146029; doi:10.3390/genes12050657)

# Grow of pure and mixed cultures

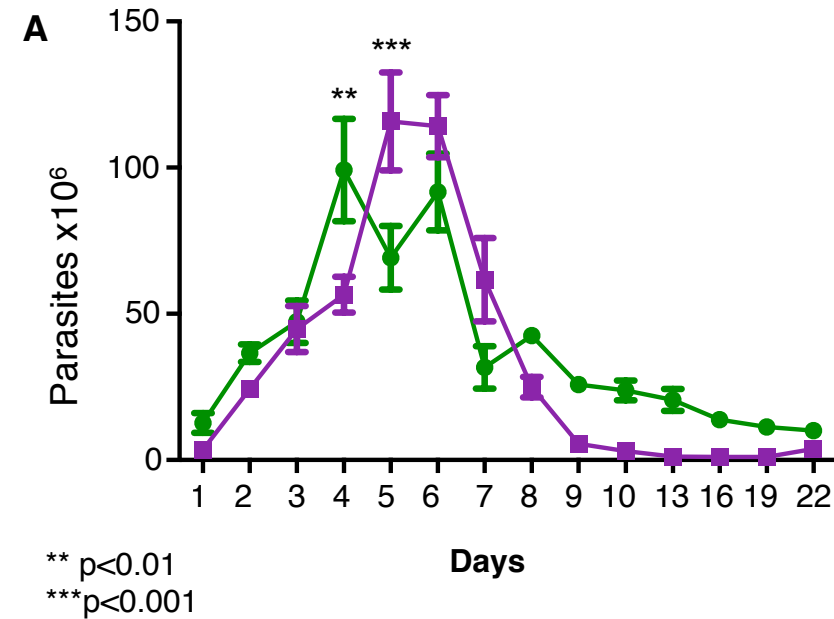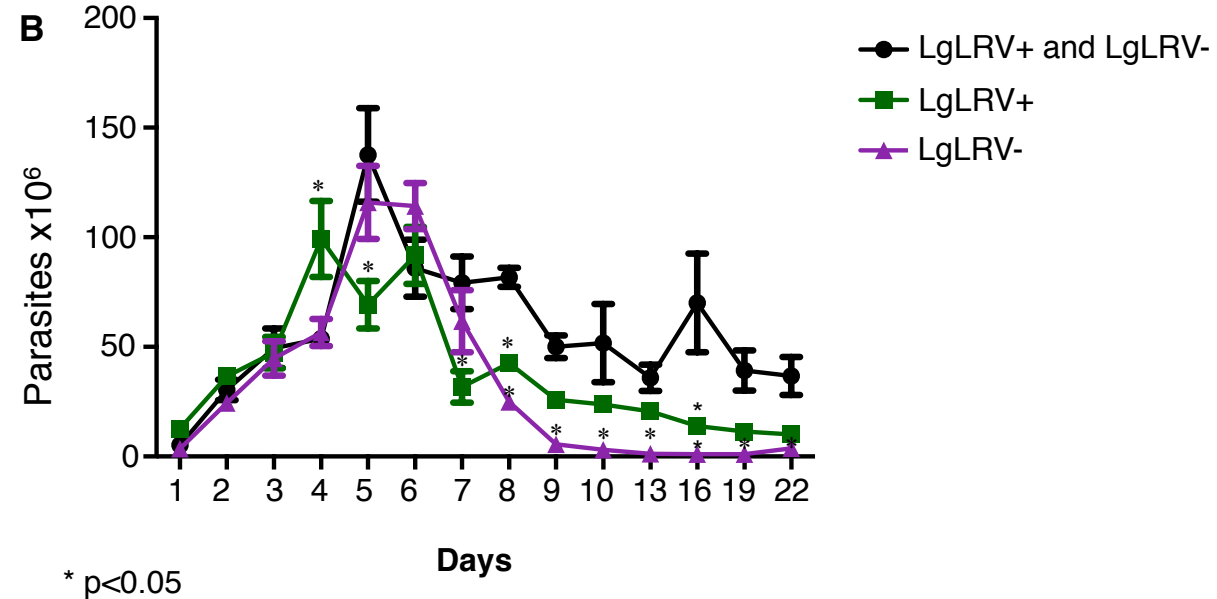

Supplement: Supplementary file 1 [file genes-12-00657-s001.zip › genes-1094199-supplementary/Figure S1.pdf]
